# Effects of succinate supplementation on rumen fermentation, microbiota, and propionate-associated metabolism *in vitro*

**DOI:** 10.3389/fmicb.2026.1836724

**Published:** 2026-06-29

**Authors:** Wenyi Ren, Xiangde Zhu, Yuchen Cheng, Xiaofeng Xu, Lili Zhang

**Affiliations:** College of Animal Science and Technology, Ningxia University, Yinchuan, China

**Keywords:** bacterial community composition, *in vitro* fermentation, rumen propionate, succinate, targeted metabolomics

## Abstract

**Introduction:**

Succinate is an intermediate in the ruminal succinate-propionate pathway, but its direct effects as an exogenous additive on rumen fermentation, bacterial community structure, and energy-related metabolites remain insufficiently characterized. This study evaluated whether graded succinate supplementation changes *in vitro* rumen fermentation and propionate-associated microbial and metabolite profiles.

**Methods:**

Rumen fluid collected from three fistulated Chinese Holstein cows was pooled within each weekly fermentation run and incubated with alfalfa substrate and sodium succinate at 0, 4, 8, or 12 mmol/L. Gas-production kinetics, fermentation characteristics, bacterial community composition, targeted energy metabolites, and correlation networks were analyzed.

**Results:**

Succinate supplementation altered gas-production kinetics, with 24-h and potential gas production peaking at 8 mmol/L. Acetate, propionate, butyrate, and total VFA concentrations increased, whereas pH and NH_3_-N concentration were not significantly affected. Succinate supplementation was associated with shifts in bacterial relative abundance, including changes in *Succiniclasticum*, *Selenomonas*, *Butyrivibrio*, *Anaeroplasma*, *Pseudobutyrivibrio*, *Desulfovibrio*, *Fibrobacter*, and *Corynebacterium*. Targeted metabolomics comparing SA0 and SA3 showed FDR-significant changes in metabolites mapped to glycolysis/gluconeogenesis, pyruvate metabolism, the tricarboxylic acid cycle, and acetate metabolism.

**Discussion:**

These findings indicate that succinate supplementation was associated with increased propionate concentration and coordinated changes in bacterial taxa and targeted metabolites related to propionate-associated metabolism *in vitro*. Because methane production, metabolic flux, enzyme activity, gene expression, and absolute microbial abundance were not directly measured, the results should be interpreted as association-level evidence. Further *in vivo* studies and direct functional measurements are needed to determine whether succinate can be used reliably to modulate rumen fermentation.

## 1 Introduction

Rumen fermentation determines how dietary carbohydrates are converted into volatile fatty acids (VFAs), microbial biomass, gases, and ammonia nitrogen, and therefore has direct consequences for nutrient use in ruminants. Acetate, propionate, and butyrate provide most of the metabolizable energy absorbed from the rumen, whereas methane represents a loss of feed energy and a source of greenhouse gas emissions (Knapp et al., 2014; Gruninger et al., 2019). For this reason, non-antibiotic nutritional strategies that can shift fermentation products, particularly toward propionate formation, remain of interest in ruminant nutrition. Organic acids such as fumarate, malate, citrate, and lactate have been examined as rumen fermentation modifiers, but their effects depend on the compound, dose, diet, microbial community, and experimental system (Yang et al., 2012; El-Zaiat et al., 2019; Michalak et al., 2021).

Dicarboxylic acids are of particular interest because several of them are connected to the succinate-propionate pathway. In the rumen, fumarate can be reduced to succinate, and succinate can then be converted to propionate by succinate-utilizing bacteria (Sijpesteijn and Elsden, 1952; Blackburn and Hungate, 1963). Studies with fumarate or malate have reported changes in ruminal VFA profiles and, in some cases, lower acetate-to-propionate ratios or altered methane-related fermentation patterns (Baraz et al., 2018; Li et al., 2018). These findings suggest that intermediates linked to propionate formation may influence rumen fermentation, but they do not establish that each intermediate has the same effect when supplied directly.

Succinate itself has received less direct attention as an exogenous additive in rumen fermentation studies. This distinction is important because succinate is not only a product of fumarate reduction, but also a substrate that can be rapidly consumed by specific bacterial groups, including members of *Succiniclasticum* and *Selenomonas* (Wang et al., 2025). Therefore, supplementing succinate may affect propionate concentration, microbial composition, and intracellular energy-related metabolites in ways that differ from supplying upstream compounds such as fumarate or malate. At the same time, changes in relative bacterial abundance or metabolite concentration cannot by themselves prove metabolic flux or causal microbial function, and this limitation needs to be considered when interpreting multi-omics data from *in vitro* fermentation (Maghini et al., 2024).

The present study tested whether graded succinate supplementation alters *in vitro* rumen fermentation, bacterial community structure, and targeted energy-metabolite profiles associated with propionate production. We used an *in vitro* batch culture system with alfalfa substrate and four succinate levels (0, 4, 8, and 12 mmol/L), and combined gas-production measurements, fermentation indices, 16S rRNA sequencing, targeted metabolomics, and correlation analysis. We hypothesized that succinate supplementation would be associated with increased propionate concentration and coordinated changes in succinate- or propionate-associated bacteria and metabolites. The study was designed to provide association-level evidence for succinate-mediated changes in rumen fermentation and to identify questions that require direct functional validation in future work.

## 2 Materials and methods

### 2.1 Experimental design

Three Chinese Holstein dairy cows (body weight: 500 ± 23.5 kg) equipped with permanent rumen fistulas were used as rumen fluid donors. The study was approved by the Animal Ethics Committee of Ningxia University (NXU20210420). All donor cows received the same lactation diet throughout the experiment (Table 1). Rumen fluid was collected through the fistula approximately 3 h after morning feeding. Equal volumes of rumen fluid from the three donor cows were pooled within each weekly run, filtered under CO_2_, and transported to the laboratory under pre-warmed anaerobic conditions at 39 °C.

**Table 1:** Composition and nutrient levels of diets (DM basis).

Ingredient	Content %	Nutrient level	Content
Whole-plant corn silage	45.06	CP^b^, %	11.69
Alfalfa silage	4.12	NE_L_^c^ (MJ/kg)	5.94
Alfalfa^a^	10.32	Ca, %	0.67
Flaked corn	22.50	P, %	0.28
Pelleted sugar beet pulp	2.00	NDF^d^, %	30.74
Brewer’s grains	1.53	ADF^e^, %	17.80
Concentrate feed	14.3		
Sodium bicarbonate	0.17		
Total	100.00		

a Alfalfa contained (DM basis): 91.79% DM, 13.53% CP, 1.03% EE, 7.69% Ash, 38.22% NDF, 27.57% ADF, 1.59% CP, 0.26% P.

b CP = Crude protein.

c NE_L_ = Net Energy for Lactation (Gruber et al., 2021).

d NDF = Neutral detergent fiber.

e ADF = Acid detergent fiber.

Food-grade sodium succinate (≥99%, Jiangsu Yaoju Biotechnology Co., Ltd., China) was supplemented at four levels: 0 mmol/L (SA0), 4 mmol/L (SA1), 8 mmol/L (SA2), and 12 mmol/L (SA3). The experiment was conducted in three independent weekly fermentation runs using freshly collected pooled rumen fluid. Within each run, each treatment included six calibrated 100 mL glass syringes containing 0.2 g alfalfa substrate and 30 mL buffered rumen fluid (rumen fluid:buffer = 1:2). Gas production was recorded at 3, 6, 9, 12, and 24 h. The syringes were treated as technical replicates nested within run.

For batch fermentation, each treatment was incubated in 120 mL fermentation bottles containing 0.5 g alfalfa substrate and 50 mL buffered rumen fluid. Four bottles per treatment were incubated in each weekly run, giving 12 bottle samples per treatment across the three independent runs. The fermentation bottles were incubated anaerobically at 39 °C for 24 h in a constant-temperature shaking incubator.

### 2.2 Sample collection

After 24 h of incubation, fermentation was terminated by placing bottles in ice water. Fermentation-fluid pH was measured immediately. Samples were centrifuged at 10,000 rpm for 10 min at 4 °C. For VFA analysis, 1 mL of supernatant was mixed with 0.2 mL of 25% metaphosphoric acid, incubated for 15 min, and stored at −20 °C until analysis. Additional aliquots were stored at −20 °C for NH_3_-N determination, and 2 mL samples were snap-frozen in liquid nitrogen for subsequent bacterial community and metabolomics analyses.

### 2.3 Gas production and kinetic parameters of gas production *in vitro*

Net gas production was calculated as follows:


$$\begin{array}{l} \mathrm{Net}\;\mathrm{gas}\;\text{production}\;(\mathrm{mL})=\mathrm{Gas}\;\text{production during}\;a\;\text{certain}\;\\ \text{period}\;(\mathrm{mL})-\text{Blank}\;\mathrm{gas}\;\text{production during that period}\;(\mathrm{mL}) \end{array}$$

Gas production parameters were calculated using curve-fitting software based on the model of Ørskov and McDonald (1979):


$$P=a+b\;(1-e^{-\mathrm{ct}})$$

where *P* (mL) is cumulative gas production at time *t*, *a* (mL) is gas production from the immediately soluble fraction, *b* (mL) is gas production from the insoluble but fermentable fraction, *a* + *b* (mL) represents potential gas production, *c* (h^−1^) is the fractional gas-production rate, and *t* (h) is incubation time. Negative *a* values were retained when generated by the model fit and were interpreted as mathematical intercept estimates rather than biologically meaningful gas production.

### 2.4 Determination of NH3-N and VFAs

NH_3_-N concentration was measured using the phenol-sodium hypochlorite colorimetric method as described by Coelho et al. (2025). VFAs, including acetate, propionate and butyrate, were quantified using gas chromatography on a GC-2030 system (Shimadzu, Japan) equipped with a flame-ionization detector and a capillary column (30 m × 0.32 mm × 0.25 μm film thickness).

The oven temperature was increased from 60 °C to 190 °C at 12.5 °C/min and held at 190 °C for 1 min. The injector and detector temperatures were 220 °C and 280 °C, respectively. High-purity nitrogen was used as the carrier gas, with an injection pressure of 110 kPa. The air flow rate was 200 mL/min, the hydrogen flow rate was 32 mL/min, the split ratio was 30:1, and the injection volume was 1 μL.

### 2.5 16S rRNA gene sequencing and bacterial community analysis

For bacterial community analysis, one 2 mL frozen fermentation-fluid aliquot from each selected fermentation bottle was transported on dry ice to Shanghai Biprofile Biotechnology Co., Ltd. for DNA extraction. Twelve samples per treatment were analyzed, giving 48 samples in total. DNA concentration and quality were assessed using a NanoDrop spectrophotometer and 1.2% agarose gel electrophoresis.

The V3–V4 region of the bacterial 16S rRNA gene was amplified using primers 341F (5′-CCTAYGGGRBGCASCAG-3′) and 806R (5′-GGACTACNNGGGTATCTAAT-3′) with sample-specific barcodes. PCR products were quantified using the Quant-iT PicoGreen dsDNA Assay Kit on a BioTek FLx800 microplate reader. Sequencing libraries were prepared using the Illumina TruSeq Nano DNA LT Library Prep Kit, checked on an Agilent Bioanalyzer with the Agilent High Sensitivity DNA Kit, quantified using the Promega QuantiFluor system, pooled in equimolar proportions, denatured with NaOH, and sequenced on an Illumina MiSeq platform.

Raw paired-end reads were demultiplexed according to index and barcode sequences, and barcode sequences were removed before quality control. Primer sequences were trimmed using cutadapt, and QIIME 22019.4 was used for downstream analysis. After quality filtering, denoising, paired-end read merging, and chimera removal with the DADA2 workflow, non-singleton amplicon sequence variants were retained for diversity analysis. Taxonomic assignment was performed using the QIIME 2 feature-classifier classify-sklearn naïve Bayes classifier against the Greengenes 13_8 99% OTU reference database.

The filtered dataset contained 48 samples, with 28,903 to 81,455 sequences per sample before rarefaction, with a mean sequencing depth of 58,263 sequences. Diversity analyses were performed after rarefaction to 27,457 sequences per sample. Alpha diversity indices were calculated in QIIME 2. Community structure was visualized using principal component analysis of bacterial abundance profiles and Bray-Curtis-based ordination. Treatment effects on beta diversity were tested using permutational multivariate analysis of variance (PERMANOVA) based on the Bray-Curtis distance matrix with 999 permutations.

### 2.6 Targeted energy metabolome analysis

Fermentation-fluid samples from the SA0 and SA3 groups were sent to Shanghai Biprofile Biotechnology Co., Ltd. for targeted energy-metabolite analysis. SA0 and SA3 were selected to compare the unsupplemented control with the highest succinate dose, thereby maximizing contrast for targeted pathway screening. The ion-peak table contained 12 SA0 samples and 12 SA3 samples for the metabolomics comparison.

Targeted metabolites involved in the tricarboxylic acid cycle, glycolysis, the pentose phosphate pathway, pyruvate metabolism, and propionate-associated metabolism were analyzed using ultra-high-performance liquid chromatography–tandem mass spectrometry (UPLC-MS/MS). For metabolite extraction, 100 μL of pre-cooled water and 800 μL of pre-cooled methanol/acetonitrile solution (1:1, v/v) were added to each sample. The mixture was vortexed, sonicated in an ice bath for 60 min, incubated at −20 °C for 1 h to precipitate proteins, and centrifuged at 16,000 × g for 20 min at 4 °C. The supernatant was collected, mixed with the internal standard L-glutamate-d5, and vacuum-dried. Before injection, the dried extract was reconstituted in 50 μL acetonitrile-water solution (1:1, v/v), centrifuged at 16,000 × g for 15 min at 4 °C, and the supernatant was used for analysis.

Metabolite separation was performed on a Shimadzu Nexera X2 LC-30 AD system using a Waters ACQUITY UPLC BEH Amide column (1.7 μm, 2.1 mm × 100 mm). The autosampler temperature was 4 °C, the column temperature was 40 °C, the flow rate was 300 μL/min, and the injection volume was 10 μL. Mobile phase A consisted of 5% acetonitrile in water containing 10 mM ammonium acetate at pH 9, and mobile phase B consisted of 95% acetonitrile in water containing 10 mM ammonium acetate at pH 9. The gradient was as follows: 95% B from 0 to 2 min, 95 to 70% B from 2 to 9 min, 70 to 30% B from 9 to 10 min, 30% B from 10 to 11 min, 30 to 95% B from 11 to 11.5 min, and 95% B from 11.5 to 15 min. Quality-control samples were prepared by mixing equal volumes from all samples. One QC sample was injected before the analytical queue, QC samples were inserted between sample groups, and one QC sample was injected at the end of the queue.

Mass spectrometric analysis was performed using a QTRAP 5500 mass spectrometer (AB SCIEX) in positive and negative electrospray ionization modes. For positive ion mode, the source temperature, ion source gas 1, ion source gas 2, curtain gas, and ion source voltage floating were set to 550 °C, 40 psi, 50 psi, 35 psi, and 5,500 V, respectively. For negative ion mode, the corresponding settings were 550 °C, 40 psi, 50 psi, 35 psi, and −4,500 V. Target ion pairs were detected using multiple-reaction monitoring. Peak areas and retention times were extracted using MultiQuant software. Metabolites were identified by comparison with authentic standards, and peak areas were normalized to L-glutamate-d5.

### 2.7 Statistical analysis

Gas production and rumen fermentation data were organized using Excel 2019 and analyzed in SAS 9.4 using PROC MIXED. Treatment was included as a fixed effect, and weekly fermentation run was included as a random effect. Syringe or bottle measurements were treated as technical replicates nested within run. Gas production included six syringes per treatment in each of three runs, giving 18 syringe observations per treatment. Fermentation, microbiome, and metabolomics measurements were based on 12 bottle-derived samples per treatment when the corresponding assay was performed. Targeted metabolomics compared SA0 and SA3 only, with 12 samples per group.

Linear and quadratic contrasts were used to evaluate dose responses. Model residuals were inspected for normality and homogeneity of variance before inference. Microbial alpha diversity and relative-abundance data were analyzed using non-parametric tests when distributional assumptions were not met. For metabolomics, differential metabolites between SA0 and SA3 were screened using fold-change and *p*-value criteria, and *p* values were adjusted using the Benjamini-Hochberg false-discovery-rate method. Spearman correlation networks among fermentation parameters, bacterial taxa, and metabolites were constructed in R using igraph with *P*-adj < 0.05 and |r| > 0.7 and visualized in Cytoscape 3.9.1. Correlations were interpreted as associations rather than causal interactions. *p* < 0.05 and FDR-adjusted q < 0.05 were considered significant, and 0.05 ≤ *p* < 0.1 was considered a tendency.

## 3 Results

### 3.1 Effect of succinate on rumen fermentation *in vitro*

As shown in Table 2, 24-h gas production and potential gas production (a + b) showed significant linear and quadratic responses to increasing succinate concentration (*p* < 0.05), with the highest values observed in the SA2 group (8 mmol/L). These results indicate that succinate supplementation altered gas-production kinetics, with the strongest 24-h and potential gas-production responses observed at 8 mmol/L.

**Table 2:** Effects of different concentrations of succinate on *in vitro* gas production and gas production kinetic parameters.

Items	Treatment				SEM	*P*-value		
	SA0	SA1	SA2	SA3		Treatment	Linear	Quadratic
24 h gas production, mL	39.27	44.27	47.6	45.43	3.13	0.09	0.04	0.04
a, mL	−2.39	0.17	0.80	2.09	1.89	0.15	0.02	0.26
b, mL	46.09^b^	48.62^ab^	51.51^a^	48.09^ab^	1.70	0.04	0.15	0.01
a + b, mL	43.70^b^	48.79^ab^	52.31^a^	50.18^a^	2.61	0.02	0.01	0.01
c, %•h ^−1^	0.10	0.10	0.10	0.10	0.01	0.98	0.96	0.74

SA0 = 0 mmol/L, SA1 = 4 mmol/L, SA2 = 8 mmol/L, SA3 = 12 mmol/L. Different lowercase letters within a row indicate significant differences (*P* < 0.05); the same letter or no letter indicates no significant difference (*P* > 0.05). a represents the rapid gas production (mL); b represents the slow gas production (mL); c is the gas production rate constant of b (%•h − 1); (a + b) is the potential gas production (mL).

As shown in Table 3, succinate supplementation had no significant effect on fermentation-fluid pH or NH_3_-N concentration (*p* > 0.05). With increasing succinate concentration, acetate, propionate, and total volatile fatty acid (TVFA) concentrations increased (*p* < 0.05). The simultaneous increase in acetate and propionate suggests a broader stimulation of fermentative activity rather than a simple shift from acetate toward propionate.

**Table 3:** Effects of different concentrations of succinate on rumen fermentation parameters *in vitro* for 24 h.

Items	Treatment				SEM	*P*-value		
	SA0	SA1	SA2	SA3		Treatment	Linear	Quadratic
pH	7.12	7.14	7.15	7.13	0.05	0.95	0.87	0.56
NH_3_-N, mg/100 mL	13.61	13.96	13.48	13.78	0.62	0.88	0.99	0.96
Acetate, mmol/L	43.76^b^	47.69^a^	48.86^a^	48.97^a^	1.77	0.02	*P* < 0.01	0.05
Propionate, mmol/L	14.36^c^	18.09^b^	19.66^b^	23.08^a^	1.03	*P* < 0.01	*P* < 0.01	*P* < 0.01
Butyrate, mmol/L	4.15^b^	4.66^ab^	4.74^ab^	5.16^a^	0.28	0.01	*P* < 0.01	0.42
Acetate/Propionate	3.05^a^	2.64^b^	2.53^b^	2.13^c^	0.06	*P* < 0.01	*P* < 0.01	0.01
Total VFAs, mmol/L	62.27^c^	70.44^b^	73.25^ab^	77.21^a^	3.00	*P* < 0.01	*P* < 0.01	0.07

SA0 = 0 mmol/L, SA1 = 4 mmol/L, SA2 = 8 mmol/L, SA3 = 12 mmol/L. Different lowercase letters within a row indicate significant differences (*P* < 0.05); the same letter or no letter indicates no significant difference (*P* > 0.05).

### 3.2 Effect of succinate on bacterial flora structure in rumen *in vitro*

**Figure 1:**
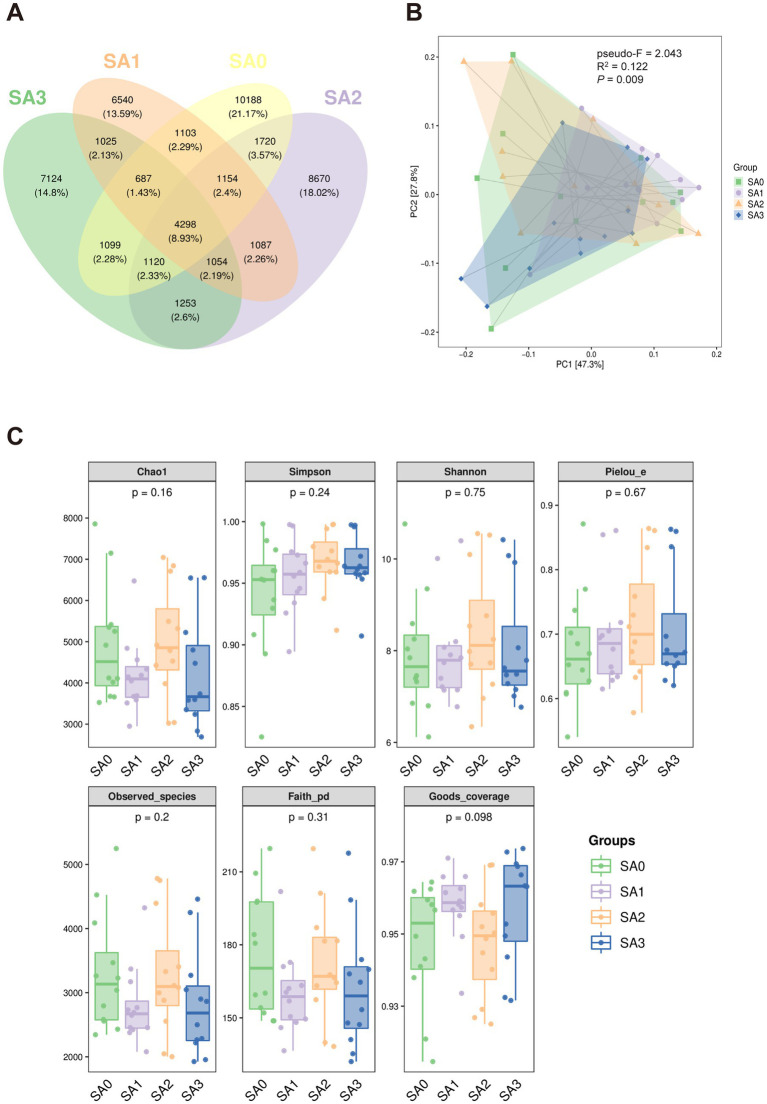
Succinate altered the bacterial community structure of *in vitro* rumen fermentation fluid. **(A)** Venn diagram of retained bacterial sequence features. **(B)** Bacterial community ordination by PCA. **(C)** Alpha diversity indices. SA0 = 0 mmol/L, SA1 = 4 mmol/L, SA2 = 8 mmol/L, SA3 = 12 mmol/L.

As shown in Figure 1A, a total of 48,122 bacterial sequence features were retained across all groups after rarefaction, with 4,298 features shared among the four groups. The numbers of features unique to the SA0, SA1, SA2, and SA3 groups were 10,188, 6,540, 8,670, and 7,124, respectively. In Figure 1B, the SA0 group separated from the SA1 and SA3 groups, whereas the SA1, SA2, and SA3 groups were closer and partially overlapped, suggesting treatment-associated community differences. Alpha diversity indices, including Chao1, observed species, Shannon, Simpson, Faith’s PD, and Pielou’s evenness, did not differ significantly among groups (*p* > 0.05; Figure 1C).

At the phylum level, 33 bacterial phyla were identified in the fermentation-fluid samples. Proteobacteria, Firmicutes, and Bacteroidetes had the highest mean relative abundances (33.95, 32.23, and 29.47%, respectively; Table 4). The relatively high Proteobacteria abundance may reflect the *in vitro* incubation environment and should be interpreted in that context. Bray-Curtis PERMANOVA indicated that bacterial community composition differed among succinate treatments (*p* = 0.009). With increasing succinate concentration, the relative abundances of Firmicutes, Tenericutes, and Fibrobacteres increased linearly (*p* < 0.05), whereas Actinobacteria decreased linearly and quadratically (*p* < 0.05). At the genus level, 44 genera were identified, and genera with relative abundance >1% were considered dominant. *Prevotella*, *Shigella*, *Succiniclasticum*, and *Streptococcus* had relatively high mean abundances (18.66, 6.84, 6.13, and 5.53%, respectively; Table 5). The assignment of Shigella in rumen samples is unusual and may reflect the resolution limits of 16S rRNA gene classification and the Greengenes database; therefore, this taxon was not interpreted as direct evidence of Shigella colonization. Several genera whose abundances changed with succinate, including *Succiniclasticum*, *Selenomonas*, *Butyrivibrio*, *Anaeroplasma*, *Pseudobutyrivibrio*, *Desulfovibrio*, and *Fibrobacter*, may participate directly or indirectly in carbohydrate fermentation and VFA-associated metabolism, but genus-level relative abundance alone cannot confirm functional propionate production.

**Table 4:** Effects of succinate on phylum-level bacterial relative abundance in fermentation fluid (%).

Phylum level	Treatment				SEM	*P-*value		
	SA0	SA1	SA2	SA3		Treatment	Linear	Quadratic
Proteobacteria	34.81	37.02	31.13	32.84	7.55	0.88	0.62	0.82
Firmicutes	29.03	31.26	31.49	37.14	3.97	0.22	0.05	0.98
Bacteroidetes	30.19	27.76	33.4	26.52	4.28	0.40	0.70	0.56
Actinobacteria	3.80^a^	1.52^b^	1.06^b^	0.63^b^	0.69	*P* < 0.01	*P* < 0.01	*P* < 0.01
Tenericutes	0.58^b^	0.61^b^	0.81^a^	0.78^a^	0.08	0.01	*P* < 0.01	0.17
SR1	0.60	0.49	0.61	0.44	0.09	0.20	0.22	0.95
TM7	0.27	0.28	0.35	0.31	0.05	0.43	0.23	0.40
Fusobacteria	0.01	0.32	0.38	0.32	0.22	0.36	0.17	0.13
Fibrobacteres	0.05	0.15	0.21	0.42	0.17	0.17	0.03	0.85
Spirochaetes	0.11	0.21	0.14	0.22	0.07	0.31	0.23	0.60

SA0 = 0 mmol/L, SA1 = 4 mmol/L, SA2 = 8 mmol/L, SA3 = 12 mmol/L. Different lowercase letters within a row indicate significant differences (*P* < 0.05); the same letter or no letter indicates no significant difference (*P* > 0.05).

**Table 5:** Effects of succinate on genus-level bacterial relative abundance in fermentation fluid (%).

Genus level	Treatment				SEM	*P-*value		
	SA0	SA1	SA2	SA3		Treatment	Linear	Quadratic
*Prevotella*	20.30	16.57	22.26	15.50	3.02	0.10	0.38	0.69
*Shigella*	8.97	4.18	5.56	8.66	2.92	0.29	0.96	0.07
*Succiniclasticum*	4.96	6.66	5.95	6.93	0.9	0.14	0.08	0.30
*Streptococcus*	6.84^a^	3.18^b^	5.59^ab^	6.49^a^	1.36	0.04	0.77	0.04
*Campylobacter*	0.67^b^	5.34^ab^	3.89^ab^	7.93^a^	2.33	0.03	0.01	0.34
*Selenomonas*	1.84^b^	2.32^ab^	2.61^ab^	3.11^a^	0.44	0.04	*P* < 0.01	0.42
*Corynebacterium*	3.55^a^	1.23^b^	0.77^b^	0.32^b^	0.68	*P* < 0.01	*P* < 0.01	*P* < 0.01
*Butyrivibrio*	1.01	1.25	1.42	1.73	0.26	0.06	*P* < 0.01	0.53
*Anaeroplasma*	0.50^b^	0.53^b^	0.70^a^	0.68^a^	0.07	0.01	*P* < 0.01	0.17
*YRC22*	0.50	0.49	0.60	0.49	0.08	0.48	0.79	0.39
*Ruminococcus*	0.27	0.38	0.28	0.37	0.10	0.59	0.56	0.77
*Pseudobutyrivibrio*	0.24^b^	0.26^ab^	0.36^a^	0.37^a^	0.05	0.03	0.00	0.30
*Clostridium*	0.22	0.33	0.27	0.36	0.12	0.70	0.38	0.73
*Oscillospira*	0.18	0.33	0.23	0.28	0.07	0.15	0.34	0.18
*Snodgrassella*	0.12	0.35	0.30	0.24	0.1	0.13	0.34	0.03
*Coprococcus*	0.24	0.29	0.22	0.25	0.08	0.83	0.92	0.84
*Fusobacterium*	0.01	0.31	0.37	0.30	0.22	0.36	0.18	0.13
*CF231*	0.29	0.28	0.21	0.20	0.08	0.55	0.16	0.64
*Desulfovibrio*	0.09	0.23	0.24	0.32	0.11	0.21	0.04	0.32
*Fibrobacter*	0.05	0.15	0.21	0.42	0.17	0.16	0.03	0.85

SA0 = 0 mmol/L, SA1 = 4 mmol/L, SA2 = 8 mmol/L, SA3 = 12 mmol/L. Different lowercase letters within a row indicate significant differences (*P* < 0.05); the same letter or no letter indicates no significant difference (*P* > 0.05).

### 3.3 Effects of succinate on energy metabolome of rumen microorganisms *in vitro*

**Figure 2:**
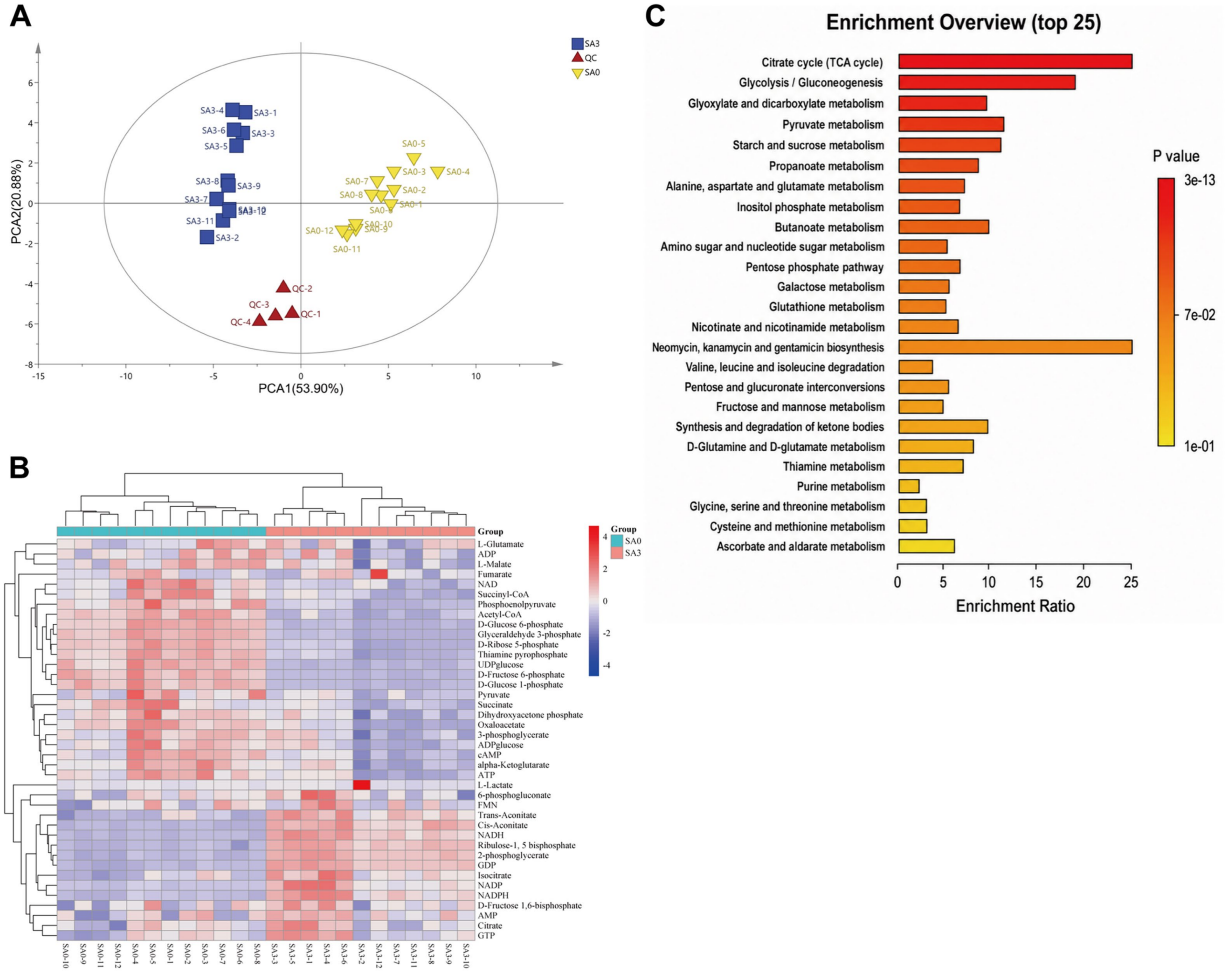
Succinate was associated with changes in targeted energy metabolites of rumen microorganisms *in vitro*. **(A)** Targeted metabolite PCA analysis. **(B)** Hierarchical clustering of targeted energy metabolites. **(C)** KEGG pathway enrichment analysis of differential metabolites. SA0 = 0 mmol/L; SA3 = 12 mmol/L.

Figure 2A shows separation between SA0 and SA3 samples in the targeted metabolite PCA, indicating differences in measured energy-metabolite profiles between the control and highest succinate dose. In the hierarchical clustering heatmap (Figure 2B), SA0 and SA3 samples clustered largely by treatment, suggesting treatment-associated differences in metabolite abundance.

A targeted panel of 40 energy metabolites was analyzed. Differential metabolites between SA0 and SA3 were screened using fold-change and FDR-adjusted significance criteria (Table 6). Compared with SA0, SA3 showed lower succinate and several glycolytic intermediates, including D-glucose-6-phosphate, fructose-6-phosphate, and phosphoenolpyruvate, whereas metabolites including NADPH, NADP, GDP, trans-aconitic acid, and 2-phosphoglycerate were increased. The decrease in measured succinate despite exogenous supplementation may indicate rapid utilization of succinate or incorporation into downstream fermentation pathways during the 24-h incubation.

**Table 6:** *In vitro* rumen fermentation broth energy differential metabolites by UPLC-MS/MS-MRM.

Metabolite name	Molecular formula	KEGG	SA0 vs. SA3		
			FC	FDR	Direction
Acetyl-CoA	C_23_H_38_N_7_O_17_P_3_S	C00024	0.44	<0.001	↓
Ribose-5-Phosphate	C_5_H_11_O_8_P	C00117	0.59	<0.001	↓
Fructose-6-Phosphate	C_6_H_13_O_9_P	C00085	0.26	<0.001	↓
Glucose-1-Phosphate	C_6_H_13_O_9_P	C00103	0.26	<0.001	↓
D-glucose-6-phosphate	C_6_H_13_O_9_P	C00092	0.49	<0.001	↓
Glyceraldehyde 3-Phosphate	C_3_H_7_O_6_P	C00661	0.44	<0.001	↓
Phosphoenolpyruvate	C_3_H_5_O_6_P	C00074	0.6	<0.001	↓
Thiamine pyrophosphate	C_12_H_19_C_l_N_4_O_7_P_2_S	C00068	0.65	<0.001	↓
Uridine diphosphate glucose	C_15_H_24_N_2_O_17_P_2_	C00029	0.39	<0.001	↓
3-phosphoglycerate	C_3_H_7_O_7_P	C00197	0.87	0.006	↓
Adenosine diphosphate glucose	C_16_H_25_N_5_O_15_P_2_	C00498	0.84	0.002	↓
Alpha-ketoglutaric acid	C_5_H_6_O_5_	C00026	0.83	0.001	↓
ATP	C_10_H_16_N_5_O_13_P_3_	C00002	0.81	<0.001	↓
cAMP	C_10_H_12_N_5_O_6_P	C00575	0.83	<0.001	↓
Dihydroxyacetone phosphate	C_3_H_7_O_6_P	C00111	0.81	<0.001	↓
L-malic acid	C_4_H_6_O_5_	C00149	0.79	0.016	↓
Oxaloacetate	C_4_H_4_O_5_	C00036	0.68	<0.001	↓
Pyruvate	C_3_H_4_O_3_	C00022	0.79	0.013	↓
succinate	C_4_H_6_O_4_	C00042	0.73	0.001	↓
NAD	C_21_H_27_N_7_O_14_P_2_	C00003	0.7	<0.001	↓
Succinyl-CoA	C_25_H_40_N_7_O_19_P_3_S	C00091	0.73	<0.001	↓
2-phosphoglycerate	C_3_H_7_O_7_P	C00631	2.31	<0.001	↑
Cis-Aconitate	C_6_H_6_O_6_	C00417	4.87	<0.001	↑
Guanosine diphosphate	C_10_H_15_N_5_O_11_P_2_	C00035	3.09	<0.001	↑
NADH	C_21_H_29_N_7_O_14_P_2_	C00004	2.45	<0.001	↑
NADP	C_21_H_29_N_7_O_17_P_3_	C00006	1.89	<0.001	↑
NADPH	C_21_H_30_N_7_O_17_P_3_	C00005	1.73	<0.001	↑
Ribulose 1,5-diphosphate	C_5_H_12_O_11_P_2_	C01182	3.87	<0.001	↑
Trans-aconitic acid	C_6_H_6_O_6_	C02341	1.53	<0.001	↑
Isocitrate	C_6_H_8_O_7_	C00311	1.39	0.002	↑

FC, fold change; FDR q-value, Benjamini-Hochberg-adjusted *P* value. Arrows indicate the direction of change in SA3 relative to SA0: ↓, lower abundance; ↑, higher abundance.

Differential energy metabolites were imported into MetaboAnalyst 5.0 for pathway enrichment analysis. The enriched pathways included the TCA cycle, glycolysis/gluconeogenesis, pyruvate metabolism, acetate metabolism, and related pathways (Figure 2C). Because pathway enrichment was based on a targeted panel and a two-group comparison, the results were interpreted as pathway-level indications rather than metabolome-wide evidence.

### 3.4 Analysis of correlation between fermentation parameters and bacteria, intermediates related to propionate production and bacteria genera related to propionate production

**Figure 3:**
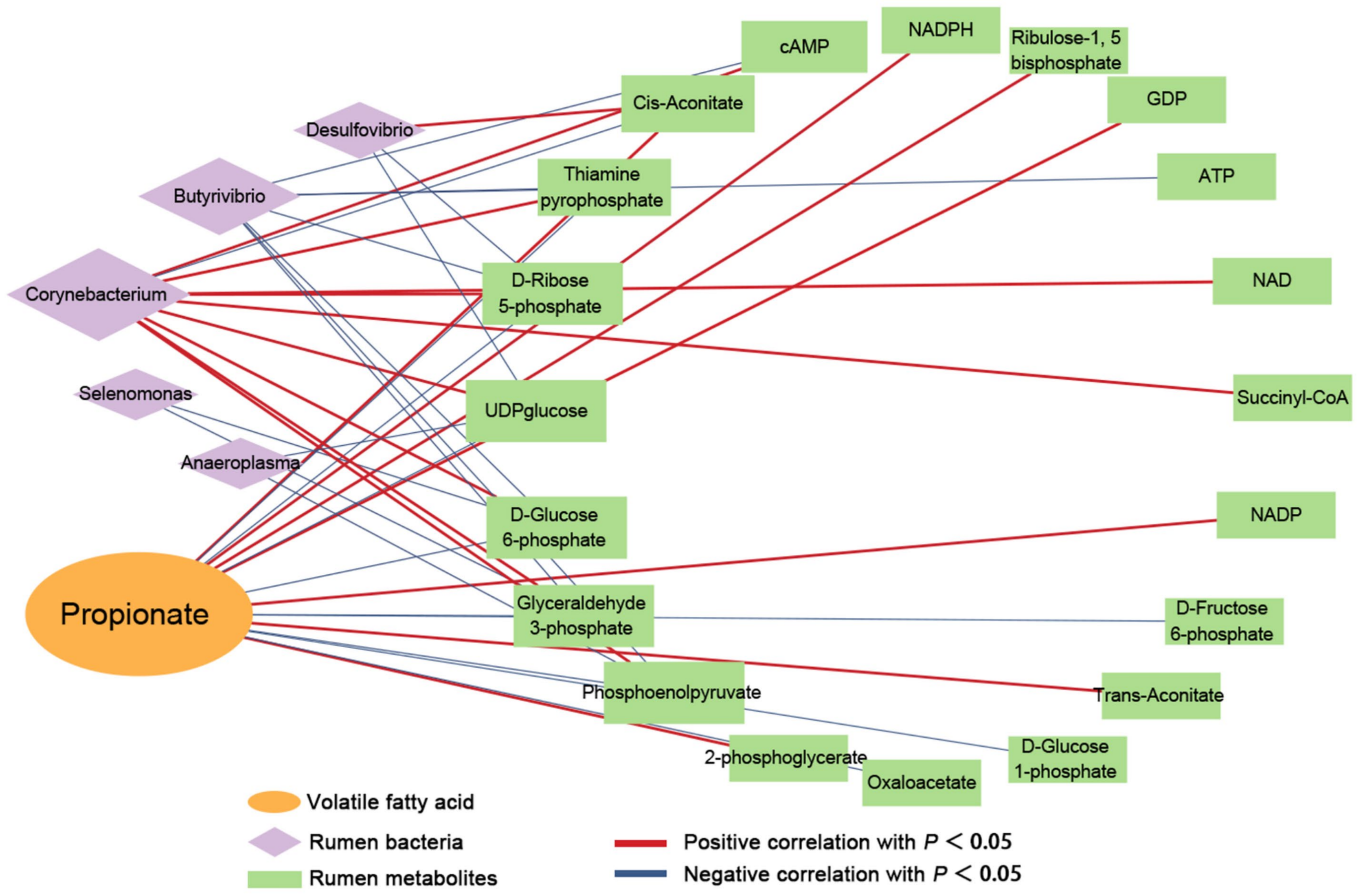
Correlation network among fermentation parameters, bacterial genera, and targeted metabolites in rumen fermentation fluid. Edges represent Spearman correlations meeting |r| > 0.7 and *P*-adj < 0.05.

Fermentation parameters, bacterial taxa, and metabolites were analyzed using Spearman correlation analysis, and associations with |r| > 0.7 and *P*-adj < 0.05 were visualized as a network (Figure 3). Propionate was associated with several differential metabolites, and Corynebacterium, *Butyrivibrio*, *Selenomonas*, *Desulfovibrio*, and *Anaeroplasma* showed correlations with targeted metabolites. Propionate and selected bacterial genera were negatively correlated with glyceraldehyde-3-phosphate, D-glucose-6-phosphate, and phosphoenolpyruvate, and positively correlated with NADPH, GDP, NADP, ribulose-1,5-bisphosphate, trans-aconitic acid, and 2-phosphoglycerate. These correlations indicate coordinated variation among fermentation products, bacterial relative abundance, and targeted metabolites, but do not establish causal interactions.

## 4 Discussion

This study showed that, in an alfalfa-based *in vitro* rumen fermentation system, exogenous succinate significantly altered the distribution of fermentation products and enhanced overall fermentative activity. The most prominent changes were an increase in propionate concentration and a decrease in the acetate-to-propionate ratio. This result is consistent with the classical understanding that succinate is an important intermediate in ruminal propionate production (Trautmann et al., 2023; Zhang et al., 2023). Succinate can be further converted to propionate by some rumen microorganisms (Daudet et al., 2026). Therefore, the increase in propionate with succinate supplementation in this study indicates that exogenous succinate most likely entered metabolic processes related to microbial propionate production in the rumen.

Notably, the succinate concentration detected after 24 h in the highest supplementation group was lower than that in the control group. At first glance, this appears inconsistent with the expectation of exogenous succinate supplementation. However, this phenomenon instead provides indirect support for the possibility that succinate was rapidly utilized or converted. If exogenous succinate had not been effectively used by microorganisms, its residual concentration would theoretically have remained higher in the high-dose group. In contrast, the decrease in succinate concentration in the SA3 group suggests that it may have been continuously consumed during incubation and further directed toward propionate or other downstream metabolites. Therefore, in the present system, succinate was more likely to act as an active metabolic intermediate than as an exogenous substrate that simply accumulated (Bai et al., 2026).

From the perspective of VFA composition, the effect of succinate was not simply to redirect acetate formation toward propionate formation. In this study, acetate, butyrate and total VFA concentrations also increased with succinate supplementation, whereas pH and NH_3_-N concentration did not change significantly. This indicates that succinate not only altered VFA partitioning, but may also have increased the overall intensity of carbohydrate fermentation. As a roughage substrate, alfalfa degradation depends on the coordinated activity of fibrolytic bacteria, sugar-fermenting bacteria and organic-acid-utilizing bacteria. After exogenous succinate entered this system, it may have altered microbial cross-feeding relationships and the utilization pattern of intermediate metabolites, thereby promoting propionate accumulation while increasing total VFA production (Li et al., 2022).

Previous studies on dicarboxylic acids such as fumarate and malate have shown that organic acids related to the succinate-propionate pathway can affect ruminal VFA composition and reduce the acetate-to-propionate ratio (Yang et al., 2012; Baraz et al., 2018; Li et al., 2018). Unlike these upstream organic acids, the present study directly examined the effect of succinate itself on mixed rumen microbial fermentation. Therefore, this study is closer to the key intermediate step of the propionate-production pathway and provides supplementary evidence for understanding how organic acids regulate ruminal carbon-flow partitioning. In other words, the significance of this study lies not only in the observed increase in propionate, but also in suggesting that key intermediate metabolites themselves may serve as effective entry points for regulating the direction of rumen fermentation.

The stability of pH and NH_3_-N provides an important boundary for interpreting the effect of succinate. Theoretically, an increase in total VFA may lead to a decrease in pH, but pH did not change significantly in this study. This indicates that, under the conditions of the present *in vitro* buffer system and alfalfa substrate, succinate promoted VFA production without causing obvious acidification. The absence of a significant change in NH_3_-N concentration suggests that succinate had limited effects on protein degradation, ammonia-N release or nitrogen metabolic balance. Therefore, the main effect of succinate in this study was more likely concentrated on carbohydrate fermentation, reducing-power utilization and VFA partitioning rather than on nitrogen release.

The gas-production results further showed that different fermentation indices did not respond to succinate dose in exactly the same way. The 24-h gas production and potential gas production reached their highest values in the 8 mmol/L group, whereas propionate concentration increased with increasing succinate supplementation. This result suggests that the dose most favorable for gas production was not necessarily the dose most favorable for propionate accumulation. *In vitro* gas production can reflect substrate degradation and the extent of microbial fermentation, but it also includes CO2 released during buffering reactions and fermentation (Amanzougarene and Fondevila, 2020; Benchaar et al., 2024). Therefore, increased gas production cannot be simply equated with improved fermentation efficiency. Succinate at 8 mmol/L may have been more favorable for overall fermentative activity and gas release, whereas succinate at 12 mmol/L may have more clearly promoted propionate-related metabolism. This difference indicates that the evaluation of rumen fermentation modulation should not rely on a single indicator, but should comprehensively consider VFA partitioning, gas production, methane production, microbial growth and the direction of carbon flow (Yanibada et al., 2021).

The negative value of gas-production parameter a should also be explained in relation to model characteristics and substrate properties. Parameter a is commonly regarded as gas production from the rapidly soluble fraction, but in essence it is an intercept-related parameter obtained from model fitting. In substrates with high structural carbohydrate content, such as alfalfa, microbial attachment, fiber hydrolysis and the initiation of early fermentation require time. Therefore, model extrapolation near time zero may produce a negative intercept. This negative value does not indicate real negative gas production, but reflects the incomplete match between the mathematical model and the initiation process of roughage fermentation (Huang et al., 2019). By comparison, 24-h gas production and potential gas production better reflect the effect of succinate on fermentation extent in this study.

The microbial community results provide further explanation for the VFA changes. Succinate supplementation did not significantly alter alpha diversity, indicating that the overall richness and evenness of the bacterial community remained relatively stable. However, beta diversity and taxonomic composition showed clear differentiation in community structure among treatments. This phenomenon indicates that succinate did not simply increase or decrease bacterial diversity, but changed the relative proportions among specific bacterial groups. For a mixed rumen microbial system, this result is reasonable. After an exogenous intermediate metabolite enters the system, it may not necessarily cause overall fluctuation in community diversity, but it may alter the competitive relationships among bacterial groups that can utilize the substrate or are affected by its metabolism (Mu et al., 2022).

At the genus level, *Selenomonas* increased with succinate supplementation, and *Succiniclasticum* also showed a higher-abundance trend in the supplemented groups. Both are associated with succinate utilization and propionate production; therefore, their changes provide a reasonable microbiological explanation for the increase in propionate. In other words, exogenous succinate may have promoted the conversion of succinate to propionate by enriching or stimulating succinate-utilizing bacterial groups. At the same time, changes in *Butyrivibrio*, *Pseudobutyrivibrio*, *Fibrobacter*, *Anaeroplasma* and *Desulfovibrio* indicate that the effect of succinate was not limited to propionate-producing bacteria. These genera are closely related to fiber degradation, carbohydrate fermentation and VFA metabolism, helping to explain the simultaneous increases in acetate, butyrate and total VFA (Goiri et al., 2020; Wang et al., 2021; Guo et al., 2022). Therefore, the microbial results were in good agreement with the fermentation results: succinate not only affected propionate-associated bacterial groups, but also reshaped the broader structure of fermentative bacterial communities. In addition, Shigella is usually not a dominant bacterium in rumen fermentation fluid, and short-fragment 16S rRNA sequencing makes it difficult to accurately distinguish Shigella from extremely closely related members of Enterobacteriaceae (Yang et al., 2022). The Greengenes database used in this study is also relatively old and has limited taxonomic resolution.

The targeted metabolomics results further showed that succinate supplementation altered the central energy-metabolic status of rumen microorganisms. The decrease in succinate concentration in the SA3 group was one of the most noteworthy metabolic phenomena in this study. At the same time, glycolysis- and pyruvate-metabolism-related intermediates such as D-glucose-6-phosphate, fructose-6-phosphate and phosphoenolpyruvate decreased, whereas metabolites or cofactors such as 2-phosphoglycerate, cis-aconitate, GDP, NADP and NADPH increased. These changes indicate that succinate supplementation did not affect only a single final product, but may have caused readjustment of central carbon metabolism, energy status and redox-related metabolite pools. This result is consistent with the increase in propionate and the alteration in VFA partitioning (Wang et al., 2022).

The changes in NADP and NADPH are particularly noteworthy. During anaerobic rumen fermentation, propionate production involves not only carbon-skeleton transformation, but also the allocation of reducing power. Propionate production can serve as an important sink for hydrogen and reducing equivalents. Therefore, when exogenous succinate enters the system and is further converted, it may alter the way microorganisms utilize reducing power. Although metabolic flux, hydrogen flow and key enzyme activities were not directly measured in this study, the metabolomics results suggest that the effect of succinate may involve coordinated adjustment of central metabolism and cofactor balance. This provides deeper metabolic clues for explaining how succinate affects VFA partitioning.

The pathway enrichment results showed that the differential metabolites were mainly involved in glycolysis/gluconeogenesis, pyruvate metabolism, the TCA cycle and acetate metabolism. These pathways together constitute the core network of rumen microbial energy metabolism and VFA production. In this study, changes in metabolites related to these pathways were consistent with changes in fermentation products and the microbial community. However, metabolite concentration reflects the size of the metabolite pool at a specific time point, rather than the actual rate of carbon flow through a given pathway. A decrease in the concentration of a metabolite may result from reduced production, or from faster consumption or conversion. Therefore, the metabolomics results should not be interpreted alone as indicating that a specific pathway was enhanced or weakened. Their more important value lies in the fact that, when combined with the VFA and microbial results, they jointly support the conclusion that succinate supplementation caused redistribution of central carbon metabolism (Mao et al., 2016).

The correlation network further integrated the relationships among fermentation indices, bacterial taxa and metabolites. Propionate was correlated with central metabolites such as glyceraldehyde-3-phosphate, D-glucose-6-phosphate, phosphoenolpyruvate, NADPH, GDP, NADP, ribulose-1,5-bisphosphate, trans-aconitic acid and 2-phosphoglycerate. Genera such as *Selenomonas*, *Butyrivibrio*, *Desulfovibrio* and *Anaeroplasma* were also correlated with various targeted metabolites. These results indicate that, after succinate supplementation, fermentation products, microbial communities and metabolite pools did not change independently, but showed an interrelated response pattern. This multi-level evidence strengthens the credibility of the study’s conclusion: succinate not only increased propionate concentration, but was also accompanied by systematic changes in microbial structure and energy-metabolic status (Zhang et al., 2022).

Regulation of rumen fermentation can be achieved not only by changing dietary composition or adding exogenous microorganisms, but also by supplementing key intermediate metabolites to influence carbon-flow partitioning. Succinate is located at the connecting point among carbohydrate fermentation, reducing-power balance and propionate production. Therefore, it is both a metabolic intermediate and an important entry point for observing the redistribution of carbon flow by rumen microorganisms. Compared with upstream organic acids such as fumarate and malate, direct supplementation with succinate allows a more direct examination of the response of the succinate-propionate pathway in a mixed rumen microbial system. This finding broadens the research perspective on the regulation of rumen fermentation by organic acids.

Succinate, or regulatory strategies targeting the succinate-propionate pathway, may have the potential to promote propionate production and improve VFA composition. For ruminants, increasing the proportion of propionate helps increase the supply of glucogenic precursors and may improve energy utilization efficiency. However, the results of this study still cannot be directly translated into feeding recommendations. The *in vivo* rumen environment is far more complex than an *in vitro* system, and the effect of succinate *in vivo* would be jointly influenced by feed intake, dietary structure, rumen passage rate, absorption, long-term microbial adaptation and host metabolic status. Therefore, this study is more suitable as a basis for subsequent *in vivo* trials and mechanistic validation than as direct evidence that succinate can already be used as a mature feed additive (Pressman and Kebreab, 2024).

The main limitation of this study is that the results were obtained from the endpoint of 24-h incubation and therefore cannot yet reveal the dynamic process of succinate utilization and conversion. Succinate may be rapidly consumed during the early stage of fermentation and may further affect propionate, acetate and butyrate production through microbial cross-feeding. A single time point cannot distinguish the temporal sequence among succinate production, succinate utilization and propionate accumulation. Similarly, 16S rRNA sequencing can only reflect changes in relative abundance and cannot prove increases in the absolute abundance, gene expression or actual metabolic activity of specific genera. Targeted metabolomics can show changes in metabolite pools, but cannot directly reflect metabolic flux. Therefore, future studies should combine time-course sampling, methane-emission and carbon-balance measurements, absolute microbial quantification, metagenomic or transcriptomic analyses, and isotope-tracing techniques to clarify the dynamic process by which exogenous succinate enters the propionate-production pathway and to determine the direction of carbon flow.

## 5 Conclusion

This study showed that succinate supplementation increased propionate concentration, decreased the acetate-to-propionate ratio and increased total VFA production in an alfalfa-based *in vitro* rumen fermentation system. The microbial and targeted metabolomics results further indicated that this fermentation response was closely related to changes in succinate-utilizing bacterial groups, fiber- and carbohydrate-fermenting bacterial groups, and central energy metabolites. This study extends current understanding of organic-acid regulation of rumen fermentation and suggests that key intermediate metabolites may regulate carbon-flow partitioning by reshaping microbial community structure and metabolite pools. Future verification of succinate-to-propionate flux under *in vivo* conditions, together with evaluation of its effects on methane production, energy utilization and animal performance, will help determine whether succinate can be developed as an effective nutritional strategy for regulating rumen fermentation.

## Data Availability

The raw data generated in this study can be found in the NCBI (https://www.ncbi.nlm.nih.gov), accession number PRJNA1103380. Further inquiries can be directed to the corresponding author.
